# Supplementary material for: Analysis of Spatial-Temporal Variation in Floral Volatiles Emitted from Lagerstroemia caudata by Headspace Solid-Phase Microextraction and GC–MS
Source: Molecules. 2023 Jan 4;28(2):478. doi: 10.3390/molecules28020478 (PMC9863544; doi:10.3390/molecules28020478)

Table S1 Volatile compounds emitted by *L. caudata*

NO.	Compound	RT	CAS#	Forluma	T_1[c]	T_2[c]	T_3[c]	P_1[d]	P_2[d]	P_3[d]	P_4[d]	P_5[d]
	**Total no. compounds (39[a]/32[b])**				**11[a]/11[b]**	**35[a]/29[b]**	**19[a]/17[b]**	**25[a]/20[b]**	**18[a]/15[b]**	**11[a]/10[b]**	**9[a]/7[b]**	**5[a]/4[b]**
	**FATTY ACID DERIVATIVES (14[a]/13[b])**											
	***Aldehydes* (3[a]/3[b])**											
1	Hexanal[2]	5.25	66-25-1	$C_6H_{12}O$	48.94 ± 2.62	2.76 ± 0.02	14.12 ± 1.55	nd	0.57 ± 0.15	8.02 ± 0.50	nd	nd
2	*trans*-2-Hexenal[2,4]	6.68	6728-26-3	$C_6H_{10}O$	41.01 ± 1.59	3.26 ± 0.14	16.51 ± 7.13	nd	nd	7.71 ± 2.66	nd	nd
3	Nonanal[2,4]	14.53	124-19-6	$C_9H_{18}O$	nd	nd	3.51 ± 0.14	nd	nd	nd	nd	nd
	***Alcohols* (7[a]/6[b])**											
4	Leaf alcohol[2,4]	6.73	928-96-1	$C_6H_{12}O$	nd	9.84 ± 0.42	12.87 ± 4.29	nd	nd	44.65 ± 2.69	nd	nd
5	*trans*-2-Hexen-1-ol[2,4]	7.03	928-94-9	$C_6H_{12}O$	0.61 ± 0.21	1.85 ± 0.10	14.64 ± 2.41	nd	nd	5.07 ± 1.69	nd	nd
6	1-Hexanol[2,4]	7.16	111-27-3	$C_6H_{14}O$	2.00 ± 0.29	13.87 ± 0.30	32.23 ± 2.77	nd	nd	6.75 ± 0.50	nd	nd
7	2-Heptanol[1,2,4]	8.10	543-49-7	$C_7H_{16}O$	0.46 ± 0.06	1.54 ± 0.03	0.26 ± 0.01	2.91 ± 0.16	nd	nd	nd	nd
8	1-Nonen-4-ol[3]	14.19	35192-73-5	$C_9H_{18}O$	nd	0.71 ± 0.34	0.44 ± 0.08	nd	0.90 ± 0.21	nd	nd	nd
9	2-Nonanol[1,2,4]	14.45	628-99-9	$C_9H_{20}O$	1.15 ± 0.04	20.86 ± 0.10	2.61 ± 0.07	41.30 ± 1.55	0.97 ± 0.11	2.65 ± 0.28	13.85 ± 0.20	nd
10	2-Undecanol[2,4]	20.37	1653-30-1	$C_{11}H_{24}O$	nd	0.12 ± 0.01	nd	0.17 ± 0.02	nd	nd	nd	nd
	***Ketones* (1[a]/1[b])**											
11	2-Nonanone[1,2,4]	14.08	821-55-6	$C_9H_{18}O$	0.21 ± 0.09	0.30 ± 0.05	nd	0.09 ± 0.00	nd	nd	nd	nd
	***Alkanes* (3[a]/3[b])**											
12	Hexadecane[2]	25.58	544-76-3	$C_{16}H_{34}$	0.09 ± 0.01	0.11 ± 0.01	0.34 ± 0.05	0.13 ± 0.04	0.42 ± 0.09	2.77 ± 0.93	8.56 ± 0.19	13.77±0.86
13	Octadecane[2]	28.00	593-45-3	$C_{18}H_{38}$	0.21 ± 0.03	0.13 ± 0.02	0.32 ± 0.04	0.15 ± 0.02	0.49 ± 0.12	3.60 ± 0.87	11.20 ± 1.32	19.58 ± 1.68
14	Eicosane[2]	34.59	112-95-8	$C_{20}H_{42}$	nd	0.10 ± 0.00	nd	nd	0.41 ± 0.04	nd	nd	nd
	**ISOPRENOIDS (19[a]/14[b])**											
	***Monoterpenes* (16[a]/11[b])**											

15	D-Limonene[3]	12.15	5989-27-5	$C_{10}H_{16}$	nd	0.09 ± 0.01	nd	0.08 ± 0.00	nd	nd	nd	nd
16	2-Furanmethanol[3]	13.48	23007-29-6	$C_{10}H_{18}O_2$	nd	0.06 ± 0.02	0.30 ± 0.03	nd	nd	nd	9.31 ± 0.56	nd
17	Linalool[1,2]	14.37	78-70-6	$C_{10}H_{18}O$	nd	5.34 ± 0.12	0.21 ± 0.03	nd	nd	nd	14.33 ± 1.84	nd
18	Citronellal[1,2,4]	15.97	106-23-0	$C_{10}H_{18}O$	nd	0.25 ± 0.09	nd	nd	2.53 ± 0.24	nd	nd	nd
19	Isopulegol[2,4]	16.07	7786-67-6	$C_{10}H_{18}O$	nd	0.25 ± 0.01	nd	0.44 ± 0.04	nd	nd	nd	nd
20	Lavandulol[2,4]	16.33	498-16-8	$C_{10}H_{18}O$	nd	0.60 ± 0.03	nd	0.90 ± 0.03	0.58 ± 0.06	nd	nd	nd
21	1,7-Octadien-3-ol, 2,6-dimethyl-[3]	16.75	22460-59-9	$C_{10}H_{18}O$	nd	0.67 ± 0.02	nd	0.88 ± 0.01	0.39 ± 0.05	nd	nd	nd
22	r-Cyclogeraniol[2,4]	16.80	6627-74-3	$C_{10}H_{18}O$	nd	0.64 ± 0.01	nd	0.61 ± 0.04	nd	nd	nd	nd
23	α-Terpineol[3]	17.32	98-55-5	$C_{10}H_{18}O$	nd	0.10 ± 0.00	nd	tr	nd	nd	nd	nd
24	Grandlure Ⅰ[3]	17.90	30820-22-5	$C_{10}H_{18}O$	nd	0.22 ± 0.01	nd	1.02 ± 0.55	nd	nd	nd	nd
25	Nerol[1,2,4]	18.14	106-25-2	$C_{10}H_{18}O$	nd	17.36 ± 0.92	0.98 ± 0.05	28.33 ± 0.30	nd	nd	nd	nd
26	Citronellol[1,2,4]	18.17	106-22-9	$C_{10}H_{20}O$	nd	3.31 ± 1.11	1.41 ± 0.08	3.19 ± 1.07	5.95 ± 0.48	nd	nd	nd
27	β-Citral[1,2,4]	18.53	106-26-3	$C_{10}H_{16}O$	nd	1.23 ± 0.14	nd	0.57 ± 0.01	4.71 ± 1.30	nd	nd	nd
28	Geraniol[1,2]	18.85	106-24-1	$C_{10}H_{18}O$	nd	6.32± 0.30	0.22 ± 0.02	9.70 ± 0.53	1.36 ± 0.30	nd	nd	nd
29	(E)-Citral[1,2,4]	19.37	141-27-5	$C_{10}H_{16}O$	nd	1.32 ± 0.14	nd	0.50 ± 0.00	6.161 ± .49	nd	nd	nd
30	Methylgeranate[2,4]	20.87	2349-14-6	$C_{11}H_{18}O_2$	nd	0.46 ± 0.06	nd	0.33 ± 0.02	nd	nd	nd	nd
	***Irregular terpenes* (2[a]/2[b])**											
31	p-Anisaldehyde[1,2,4]	19.04	123-11-5	$C_8H_8O_2$	nd	0.43 ± 0.07	nd	nd	34.01 ± 3.31	nd	nd	nd
32	Nerylacetone[2]	24.23	3879-26-3	$C_{13}H_{22}O$	nd	nd	0.18 ± 0.01	nd	nd	nd	nd	nd
	***Sesquiterpenes* (1[a]/1[b])**											
33	Caryophyllene[2]	23.58	87-44-5	$C_{15}H_{24}$	0.32 ± 0.01	0.26 ± 0.02	nd	0.09 ± 0.03	nd	nd	6.04 ± 2.03	nd
	**BENZENOIDS (5[a]/4[b])**											
	***Aldehydes* (1[a]/1[b])**											
34	Benzeneacetaldehyde[2,4]	12.65	122-78-1	C_8H_8O	nd	0.24 ± 0.13	0.39 ± 0.13	nd	1.26 ± 0.15	nd	nd	nd

	***Alcohols* (1[a]/1[b])**											
35	Phenylethyl alcohol[1,2,4]	14.77	60-12-8	$C_8H_{10}O$	nd	6.62 ± 0.22	2.30 ± 0.08	8.61 ± 0.46	36.71 ± 1.20	nd	nd	nd
	***Esters* (3[a]/2[b])**											
36	Diethyl phthalate[3]	27.66	84-66-2	$C_{12}H_{14}O_4$	nd	nd	nd	0.14 ± 0.02	0.54 ± 0.23	7.54 ± 2.59	19.71 ± 1.03	21.19 ± 2.63
37	Diisobutyl phthalate[2]	33.67	84-69-5	$C_{16}H_{22}O_4$	0.40 ± 0.24	tr	nd	0.11 ± 0.04	nd	11.91 ± 1.04	10.55 ± 3.58	33.85 ± 3.66
38	Dibutyl phthalate[2]	35.78	84-74-2	$C_{16}H_{22}O_4$	nd	tr	nd	0.11 ± 0.01	nd	4.22 ± 0.43	8.90 ± 0.49	11.02 ± 0.75
	OTHERS **(1[a]/1[b])**											
	***Nitrogen-containing* (1[a]/1[b])**											
39	(Z)-2-Methylbutanal oxime[2,4]	6.71	49805-56-3	$C_5H_{11}NO$	nd	nd	nd	0.23 ± 0.08	1.82 ± 0.08	nd	nd	nd

Retention times (RT) are given in minutes. Numbers represent relative contribution (mean percentage ± SE).

[1] Compounds were identified by co-chromatography with authentic standard.

[2] Compounds with a provisional name (MS library match is > 90%).

[3] Unidentified compounds (MS library match is 80%–90%).

[4] Compounds were identified for the first time in flowers of *L. caudata*.

[a] Total number of volatile compounds detected.

[b] Total number of volatile compounds identified.

[c] Different times of floral emission: T_1, midnight; T_2, 6 am; T_3, 6 pm.

[d] Different floral parts: P_1, stamen; P_2, petal; P_3, calyx; P_4, pistil; P_5, pedicel.

nd, not detected; tr, trace (< 0.05%).

Figure S1 Calibration curve of 12 standards used

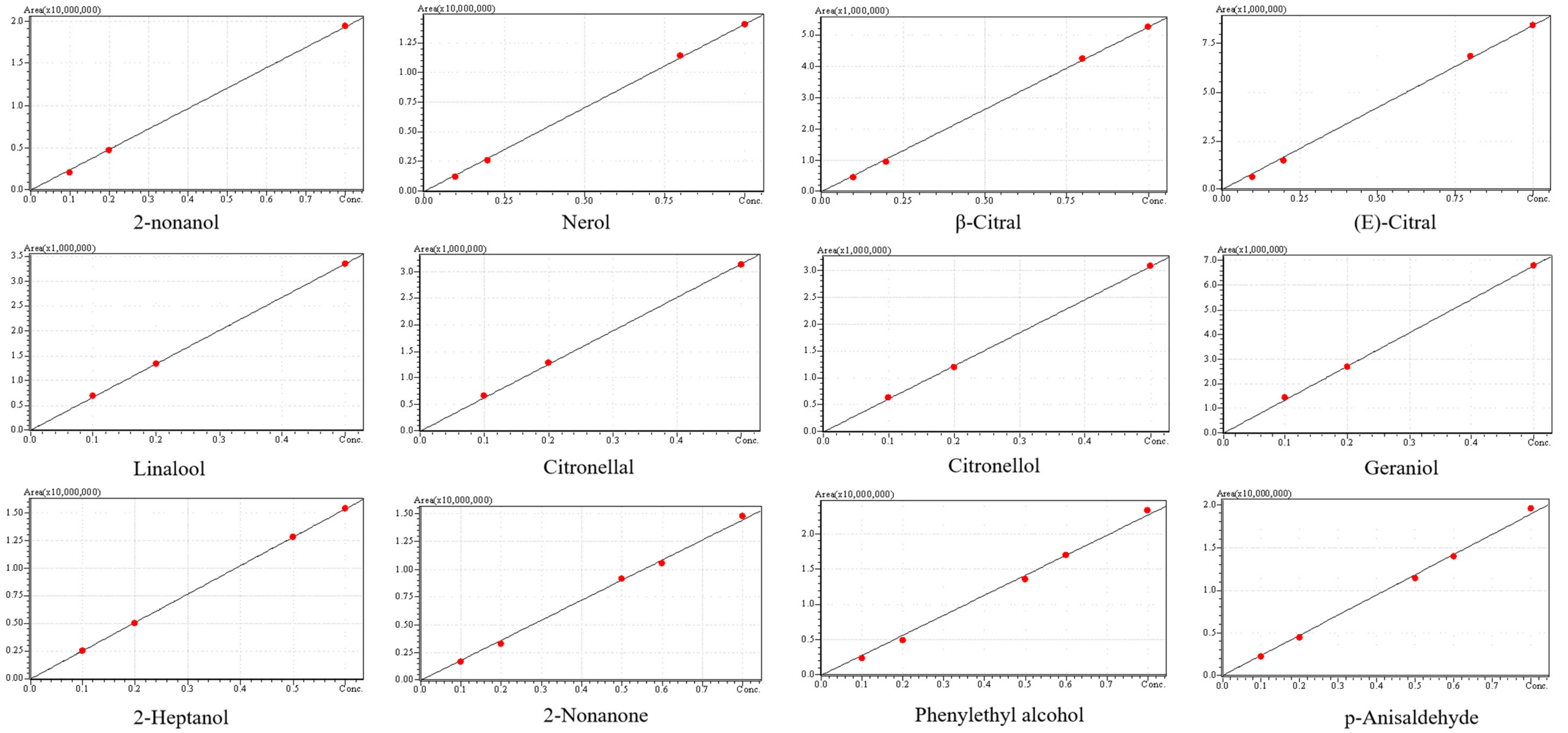

Supplement: Supplementary file 1 [file molecules-28-00478-s001.zip › molecules-2062013-supply.pdf]
